# The efficacy and safety of soluble guanylate cyclase modulation in patients with heart failure: a comprehensive meta-analysis of randomized controlled trials

**DOI:** 10.1038/s41598-024-57695-7

**Published:** 2024-03-24

**Authors:** Mehmet Emin Arayici, Hakan Gunes, Hulya Ellidokuz, Mehmet Birhan Yilmaz

**Affiliations:** 1 Department of Biostatistics and Medical Informatics, Faculty of Medicine, Dokuz Eylül University, İzmir, Turkey; 2 Department of Public Health, Faculty of Medicine, Dokuz Eylül University, İzmir, Turkey; 3 Department of Cardiology, Izmir Faculty of Medicine, University of Health Sciences, İzmir, Turkey; 4 Department of Cardiology, Faculty of Medicine, Dokuz Eylül University, Inciralti-Balcova, 35340 İzmir, Turkey

**Keywords:** Soluble guanylate cyclase, Modulator, Stimulator, Activator, Outcome, Cardiology, Cardiac device therapy

## Abstract

Soluble guanylate cyclase (sGC) modulation has been scrutinized in several disease states including heart failure (HF). Recently, it was shown that an sGC modulator improved HF-related hospitalization significantly, though, there was no benefit related to mortality. Herein, a comprehensive meta-analysis of randomized controlled trials (RCTs) for sGC modulation in HF patients was provided in agreement with the PRISMA statement. A total of 10 RCTs yielding 12 papers were included. There were 7526 patients with heart failure of each phenotype, 4253 in the sGC modulator group and 3273 in the placebo group. Use of sGC modulators in HF patients yielded no significant difference in the risk of all-cause mortality compared to placebo (RR = 0.97, 95% CI 0.88–1.08, *p* = 0.62). The use of sGC modulators was associated with a trend toward a considerable but non-significant increase in the incidence of SAEs (RR = 1.10, 95% CI 0.99–1.22, *p* = 0.07), as well as an increased incidence of hypotension and anemia. There was an overall neutral effect of sGC modulation on NT-proBNP levels, 6MWD and mortality, at a cost of slight increase in hypotension and anemia. Of note, the improvement in EQ-5D-based quality of life was significant. Hence, the benefit seems to be driven by distinctive domains of quality of life.

## Introduction

Heart failure is characterized by distorted production and/or action of nitric oxide (NO), which is a crucial signaling molecule in physiological processes, and reduced bioavailability and responsiveness to NO contribute to many diseases^1^. Soluble guanylate cyclase (sGC), as the primary receptor for NO, is a heterodimer consisting of a heme-containing subunit. It can only be induced by NO binding to its reduced Fe2+ heme moiety. In the event of loss of function, the enzyme becomes insensitive to endogenous and exogenous NO^2^.

Novel therapeutic groups, sGC-activators and sGC-stimulators, commonly called sGC-modulators, induce sGC in its NO-insensitive state, producing synergistic effects with NO^3^. The therapeutic potential of sGC modulators is greater than that of NO or NO donors. It can be attributed to the lack of uncontrolled NO release as well as the absence of tolerance development after extended treatment^4^. Hence, as a low NO-available state, heart failure with various phenotypes has been tested via sGC modulators against placebo at different settings.

Previous meta-analyses have focused on sGC modulators, revealing insights into their potential therapeutic benefits and effectiveness across heart failure. These meta-analyses aimed to consolidate and evaluate existing clinical evidence regarding sGC modulators, providing valuable insights into their effects on patient outcomes and safety profiles^5–7^. In this current research, we aimed to strengthen the evidence by conducting a more recent and comprehensive meta-analysis, encompassing a broader range of studies. Additionally, we also aimed to expand the subgroup analyses to investigate the efficacy and safety of sGC modulators more comprehensively, thus providing a more detailed examination of their impact.

## Methods

We assembled randomized controlled trials (RCTs) of sGC modulators (vericiguat, riociguat, and praliciguat as stimulators and cinaciguat as an activator) in HF patients and conducted a meta-analysis as sGC-modulators in agreement with the “Preferred Reporting Items for Systematic Reviews and Meta-Analysis (PRISMA)” statement^8^. The PRISMA checklist is available in Supplemental Table S2. The study protocol was registered in PROSPERO with the ID number CRD42021291879.

### Data sources

Two independent and qualified investigators (MEA and MBY) executed a comprehensive literature search in PubMed/Medline, Web of Science, Scopus, and the Cochrane Library up to October 12, 2023, to collect data. There was no year limitation in the search, and all studies published up to the date of the search were scrutinized. The research strategy enclosed the incorporation of key features such as Medical Subject Headings (MeSH) or keywords, text terms, and Boolean operators (AND/OR). Specifically, the search terms used were 'soluble guanylate stimulators,' 'soluble guanylate activators,' 'riociguat,' 'vericiguat,' 'praliciguat,' 'cinaciguat,' and 'heart failure,' with a publication style restriction limited to randomized controlled trials (RCT). Only English-language publications were included in the literature searches. Papers in other languages were excluded from the search strategy. The structured search strategies for the relevant databases are summarized in Supplementary Table S1.

### Study selection

In the initial search, titles and abstracts were examined independently by two investigators (MEA and MBY), and papers that were simply unrelated were omitted. The full texts of the possible papers (RCTs) that could be included in the study were examined according to the titles and abstracts. The criteria for inclusion were as follows: RCTs; adult patients (years > 18) with HF; sGC stimulators (riociguat, vericiguat or praliciguat); sGC activator (cinaciguat); placebo. Excluded from the study were reviews, studies without clinical trials, irrelevant titles, meetings, abstracts, and case reports. Primary outcome of interest was determined as all-cause mortality. The secondary outcomes included serious adverse events (SAEs) including hypotension, N-terminal natriuretic peptide levels (NT-proBNP), 6-min walking distance (6-MWD), quality of life measures and incidence of anemia. Papers that did not meet the above criteria were excluded from the study. First, duplicated articles in related databases were separated through the Mendeley data management program. Then, two independent authors (MEA and MBY) synthesized the results reported by each RCT and processed the data into a pre-prepared and structured Microsoft Excel^®^ spreadsheet.

### Data extraction and quality assessment

Baseline characteristics of studies (authors, published year, publication, and research design), characteristics of study subjects (average age, sex, comorbidities), intervention and control therapies, New York Heart Association class, left ventricular ejection fraction, follow-up time, serious adverse events, mortality, and clinical outcomes after intervention were all extracted by investigators separately from the included trials. Multiple papers reporting the same clinical study were identified. Whether the articles were taken from the same studies was scrutinized, and the most comprehensive data were chosen. When there was disagreement between the authors, it was resolved through dialogue. Allocation concealment, random sequence generation, blinding (defined as single-blind or double-blind, the process of blinding, and blinding of participants and outcomes), missing info, the possibility of reporting bias, and other biases were all included in the quality evaluation of all RCTs^9^. The evaluation was done according to the Cochrane handbook of systematic reviews of interventions 5.1.0^10^.

PICOs:

1. Population: “Patients with heart failure”
2. Intervention: “The effect of sGC stimulators and activators on patients with heart failure”
3. Comparison: “Placebo”
4. Outcomes: (1) “mortality”, (2) “quality of life”, “SAEs”, “NT-proBNP”, “6-MWD”, “KCCQ”, and “hypotension”.
5. Study: “Randomized controlled trials (RCTs)”

### Statistical analysis

The chi-squared test and I^2^ statistics were used to assess the heterogeneity of included trials. The I^2^ statistics shows the proportion of between-study variance that is due to heterogeneity rather than sampling error. Scrutinized data showing p < 0.05 and I^2^ > 50% indicated a significant level of heterogeneity^10^. If a significant level of heterogeneity was noticed, the meta-analysis was executed employing the random-effects model. Otherwise, the fixed-effects model was preferred for conducting the meta-analysis. The outcomes of mortality, SAEs, anemia, and hypotension were calculated by the risk ratio (RR) and 95% confidence interval (CI) using a fixed-effect model due to low heterogeneity. The mean change of the quality of life, N-terminal pro B type natriuretic peptide (NT-proBNP), six-minute walking distance (6MWD), and EuroQol Group 5-Dimensional Self-report Questionnaire (EQ-5D) US Index, Kansas City Cardiomyopathy Questionnaire (KCCQ) from baseline were calculated by the weighted mean difference (WMD) and mean difference (MD) with 95% CI using a random-effect and fixed-effect model according to the heterogeneity. Median and interquartile range (IQR) reported studies were converted to mean and standard deviation (SD) to conduct the meta-analysis^11^. Egger's linear regression test, schematic illustrations of funnel plots, and Begg and Mazumdar’s rank correlation test were used to quantify the possibility of publication bias^12,13^. Two-tailed p < 0.05 was deemed an indicator of statistical significance in all tests performed. The Review Manager version 5.4 (The Nordic Cochran Centre, Copenhagen, Denmark)^14^ and ProMeta3®^15^ were used for all statistical analysis and to execute the meta-analysis.

### Ethics approval

Ethical approval is not required for this study, and all techniques followed the Declaration of Helsinki.

## Results

### Search results

**Figure 1:**
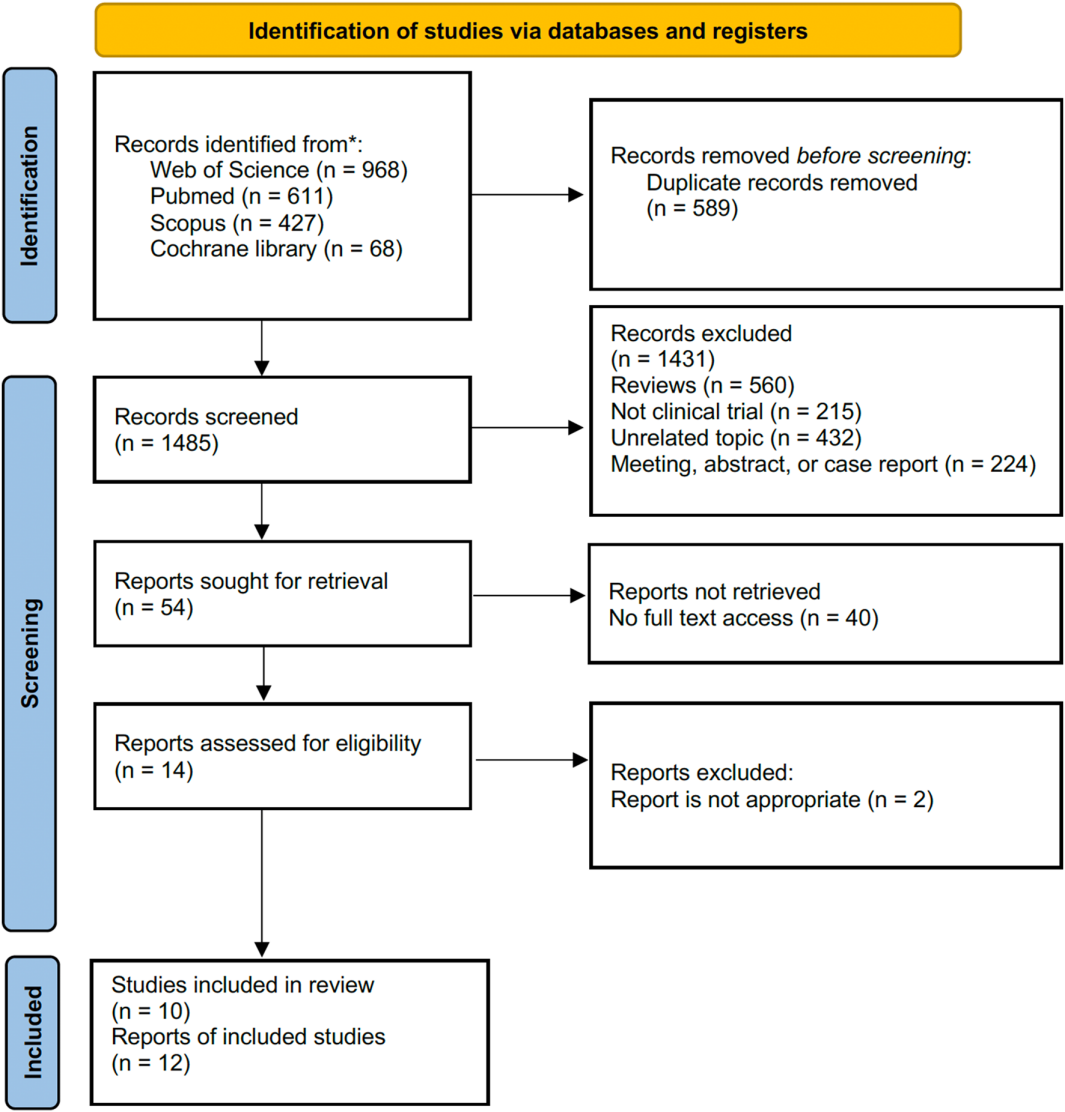
The PRISMA flow diagram showing the search strategy and included-excluded studies.

The database search resulted in a total of 2074 articles. After the exclusion of irrelevant and duplicate papers, 10 RCTs (12 papers) satisfied our inclusion criteria eventually, which were published from 2012 to 2022^16–27^. Online access to full-text journals and supplementary materials was obtained. Figure 1 illustrates the PRISMA flow diagram, the method of searching for literature and the explanations for exclusion criteria.

### Characteristics of RCTs that were included in meta-analysis

Ten RCTs (yielding 12 papers) included a total of 7,526 patients with heart failure, 4,253 in the sGC modulator group and 3,273 in the placebo group. Baseline characteristics of the studies that were included in the meta-analysis is available in Table 1. Mean age of the patients was ranging from 61 to 75 years, with a mean follow-up time ranging from 4 weeks (for safety in DILATE trial) to 47 weeks (10.8 months efficacy in VICTORIA trial). The patients had HF with either a preserved ejection fraction (HFpEF, EF > 50%) or a mildly reduced ejection fraction (HFmrEF, EF = 41–49%) or reduced ejection fraction (HFrEF, EF ≤ 40%). Vericiguat doses ranged from 1.25 to 15 mg/day, riociguat doses ranged from 0.5 to 2.5 mg 3 times/day, praliciguat dose 40 mg/day, and cinaciguat doses ranged from 50 to 100–150 ug/h.

**Table 1:** Baseline characteristics of the studies that were included in the meta-analysis.

References, first author	Study design	Year	Sample size (n) (C/T)	Interventions (sGC modulators vs. placebo)	NYHA class	LVEF (%) in T group (mean ± SD)	Mean follow-up time	Endpoints of meta-analysis
Pieske (SOCRATES-PRESERVED)	RCT	2017	93/384	Vericiguat 1.25, 2.5, 5, or 10 mg vs placebo	2–4	56.8 ± 6.25	12 wk	Mortality, SAE, EQ-5D Index Score, NT-proBNP, KCCQ, Hypotension
Gheorghiade (SOCRATES-REDUCED)	RCT	2015	92/364	Vericiguat 1.25, 2.5, 5, or 10 mg vs placebo	2–4	29.9 ± 8.4	16 wk	Mortality, SAE, NT-proBNP, Hypotension
Armstrong (VICTORIA)	RCT	2020	2526/2524	Vericiguat 2.5, 5, or 10 mg vs placebo	2–4	29.0 ± 8.3	47 wk	Mortality, SAE, Hypotension, NT-proBNP
Armstrong (VITALITY-HFpEF)	RCT	2020	262/527	Vericiguat 15 mg/d; 10 mg/d; or placebo	2–4	56.3 ± 8.1	24 wk	Mortality, KCCQ, 6MWD, NT-proBNP, SAE, Hypotension
Bonderman (phase IIb)	RCT	2013	69/132	Riociguat 0.5, 1, or 2 mg vs placebo	2–4	28.1 ± 0.8	16 wk	Mortality, SAE, EQ-5D Index Score, NT-proBNP, 6MWD, Hypotension
Bonderman (DILATE-1)	RCT	2014	11/25	Riociguat 0.5, 1, or 2 mg vs placebo	N/A	N/A	6 h, 4 weeks	Mortality, hemodynamics SAE
Udelson (CAPACITY HFpEF)	RCT	2020	91/90	Praliciguat 40 mg	2–4	N/A	12 wk	Mortality, NT-proBNP, SAE, 6MWD, KCCQ, Hypotension
Gheorgiade (phase IIb COMPOSE)	RCT	2012	22/52	CiN/Aciguat 50–100 150 ug/h	2–4	28.9 ± 5.2	5 wk	Mortality, SAE, NT-proBNP, Hypotension
Erdman (phase IIb study)	RCT	2013	51/97	CiN/Aciguat 50–600 ug/h	2–4	N/A	5 wk	Mortality, SAE, Hypotension
Dachs (haemoDYN/AMIC trial)	RCT	2022	56/58	Riociguat 0.5, 1, or 1.5 mg vs placebo	N/A	61.0 ± 6.7	26 wk	Mortality, SAE, Hypotension, 6MWD, EQ-5D Index Score

C = control group, DILATE = acute hemodyN/Amic effects of riociguat in patients with pulmoN/Ary hypertension associated with diastolic heart failure, EQ-5D = EuroQol Group 5-DmensioN/Al Self-report QuestionN/Aire, LVEF = left ventricular ejection fraction, N/A = not available, NYHA = New York Heart Association, PAH-CHD = the patients with persistent/recurrent pulmoN/Ary arterial hypertension after correction of congenital heart disease, SAE = serious adverse events, SD = standard deviation, SOCRATES-PRESERVED = soluble guanylate cyclase stimulator in heart failure patients with preserved ejection fraction, SOCRATES-REDUCED = soluble guanylate cyclase stimulator in heart failure patients with reduced ejection fraction, T = treatment group, wk = week.

**Figure 2:**
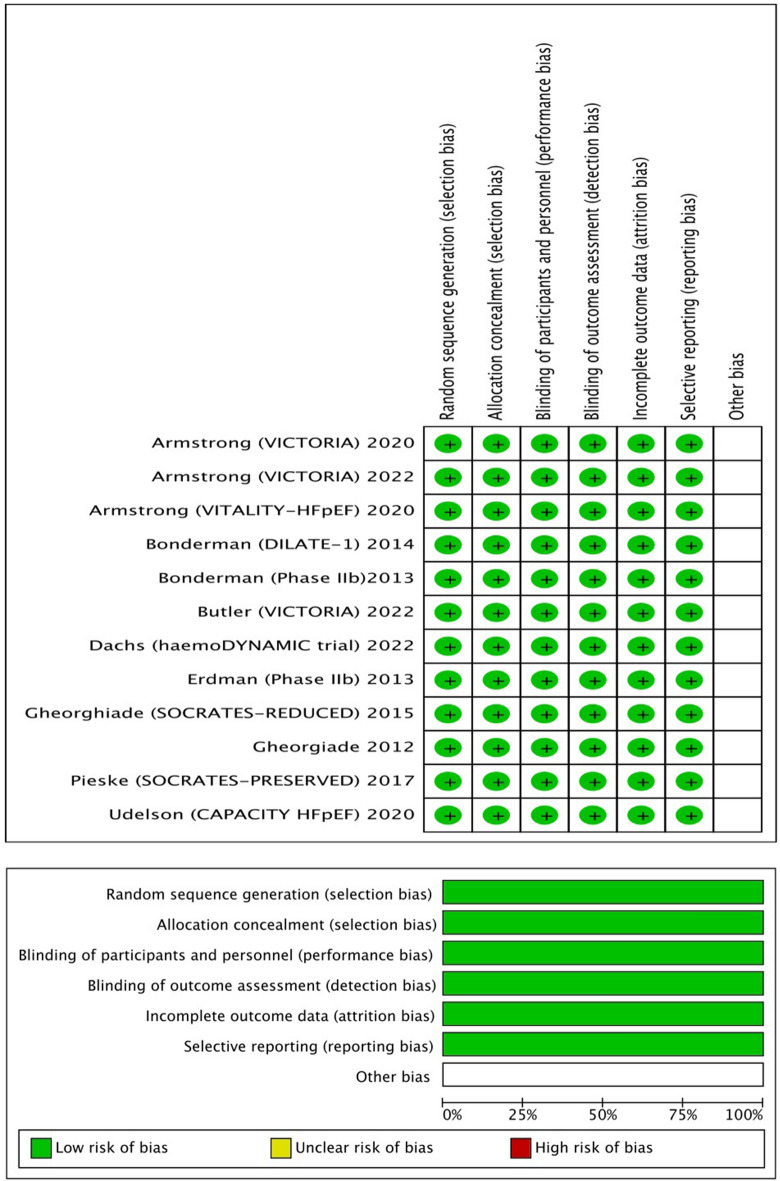
Summary and methodological quality assessment to detailed of the included RCTs.

The demographic and medication characteristics at the start of the study were reported in Table 2. Complications such as atrial fibrillation, diabetes mellitus (DM), and poor renal function were observed in some of the patients. Control and treatment arms received conventional HF therapies [diuretics, angiotensin- converting-enzyme inhibitors (ACEI), angiotensin II receptor blockers (ARB), beta-blockers, mineralocorticoid receptor antagonist (MRA)]. The Cochrane risk of bias tool was used to determine the risk of bias in 10 included trials, and the majority of items showed low risk^10^. Methodological quality assessment to detailed of the included RCTs are also presented in Fig. 2.

**Table 2:** Demographics and medication characteristics of the patients in the included RCTs.

References, first author	Mean age (years) (C/T)	Males (n%) (C/T)	Atrial fibrillation (n%) (C/T)	DM (n%) (C/T)	eGFR (mean ± SD) (C/T)	Diuretics (n%) (C/T)	ACEI (n%) (C/T)	ARB (n%) (C/T)	Beta-Blocker (n%) (C/T)	CCB (n%) (C/T)	MRA (n%) (C/T)
Pieske (SOCRATES-PRESERVED)	74 ± 9.1/73 ± 9.8	50.5/51.8	37.6/40.4	50.5/48.2	52.3 ± 20.6/55.45 ± 20.1	91.4/92.5	43.0/39.5	34.4/33.9	81.7/79.4	32.3/36.8	41.9/36.3
Gheorghiade (SOCRATESREDUCED)	67 ± 13/68 ± 12.25	79.3/80.5	32.6/34.1	44.6/48.9	57.8 ± 17.4/58.6 ± 20.0	93.5/94.5	56.5/62.6	22.8/22.8	90.2/90.1	N/A	54.3/64.3
Armstrong (VICTORIA)	67.5 ± 12 .2/67.2 ± 12.2	76/76	46.4/43.5	45.3/48.6	61.7 ± 27.3/61.3 ± 27	N/A	73.6/73.3	N/A	93.0/93.2	N/A	71.4/69.3
Armstrong (VITALITY-HFpEF)	72.8 ± 9.4/72.65 ± 9.4	53.8/50.1	60.3/62.05	46.9/44.6	56.9 ± 20.0/60.75 ± 20.9	N/A	N/A	N/A	N/A	N/A	N/A
Bonderman (phase IIb)	59 ± 40/58 ± 35	88/84	15/11.6	49/38.6	68.7 ± 2.4/69.9 ± 3.1	N/A	67/72.3	28/28.3	46/53.6	N/A	77/75.6
Bonderman (DILATE-1)	75 ± 16/70 ± 20	45/36	55/41	45/44.3	N/A	N/A	27/57	45/32.3	91/76.3	55/39	N/A
Udelson (CAPACITY HFpEF)	70.1 ± 9.0/70.7 ± 9.2	44.4/38.5	18.9/15.4	55.6/50.5	N/A	18.9/18.7	40.0/29.7	N/A	26.7/42.9	28.9/30.8	N/A
Gheorgiade (phase IIb COMPOSE)	68.7 ± 12.15/66.45 ± 12.65	90.9/80.7	68.1/44.2	36.3/21.1	N/A	81.8/84.6	54.4/65.3	N/A	59.09/53.8	N/A	N/A
Erdman (phase IIb study)	61 ± 10/62 ± 12	90.7/74.5	N/A	23.5/26.8	N/A	94.1/94.8	92.2/84.5	N/A	86.3/79.4	3.9/10.3	N/A
Dachs (haemoDYN/AMIC trial)	72.1 ± 8.5/ 70.6 ± 8.0	33.9/20.7	66.1/62.1	28.6/27.6	61.7 ± 20.1/63.4 ± 21.9	80.4/69.0	71.4/72.4	71.4/72.4	75.0/74.1	N/A	75.0/69.0
Armstrong (VICTORIA)	67.5 ± 12 .2/67.2 ± 12.2	76/76	46.4/43.5	45.3/48.6	61.7 ± 27.3/61.3 ± 27	N/A	73.6/73.3	N/A	93.0/93.2	N/A	71.4/69.3

ACEI = angiotensin-converting-enzyme inhibitor, ARB = angiotensin receptor blocker, C = control group, CCB = calcium channel blocker, DM = diabetic mellitus, eGFR = estimated glomerular filtration rate (mL/ min/1.73 m2), MRA = mineralocorticoid receptor antagonist, N/A = not available, T = treatment group.

### Outcomes of efficacy and safety analysis to overall studies

**Figure 3:**
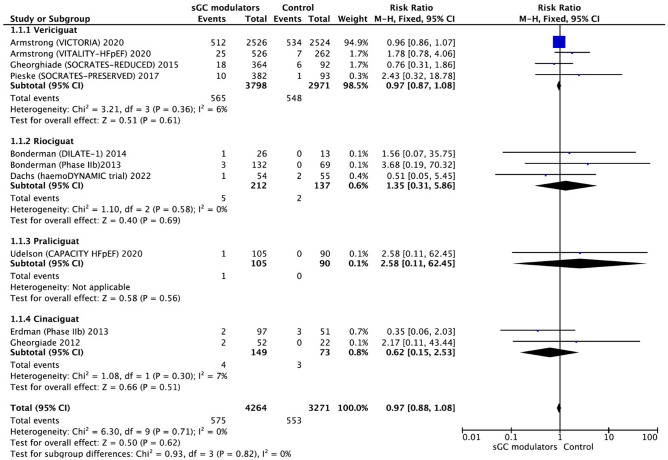
The forest plot of the effect of soluble guanylate cyclase modulators on al-cause mortality in heart failure patients compared to placebo. CI = confidence interval.

The included studies reported the efficacy of sGC modulators (riociguat, vericiguat, praliciguat as stimulators and cinaciguat as an activator) in HF patients. A total of 10 RCTs was assessed for the association between sGC modulation and all-cause mortality. The analyses of RCTs according to the fixed effect model yielded that the use of sGC modulators in HF patients resulted in no significant benefit in the risk of all-cause mortality (RR = 0.97, 95% CI 0.88–1.08, *p* = 0.62) (Fig. 3). There was no significant heterogeneity (I^2^ = 0.0%, *p* = 0.71) among the included trials, and the analysis was carried out via a fixed effect model. No significant publication bias was detected according to Egger's linear regression test and Begg and Mazumdar’s rank correlation test (Eggers’s test: *p* = 0.38; Begg’s test: *p* = 0.62) (Supp. Fig. S1).

**Figure 4:**
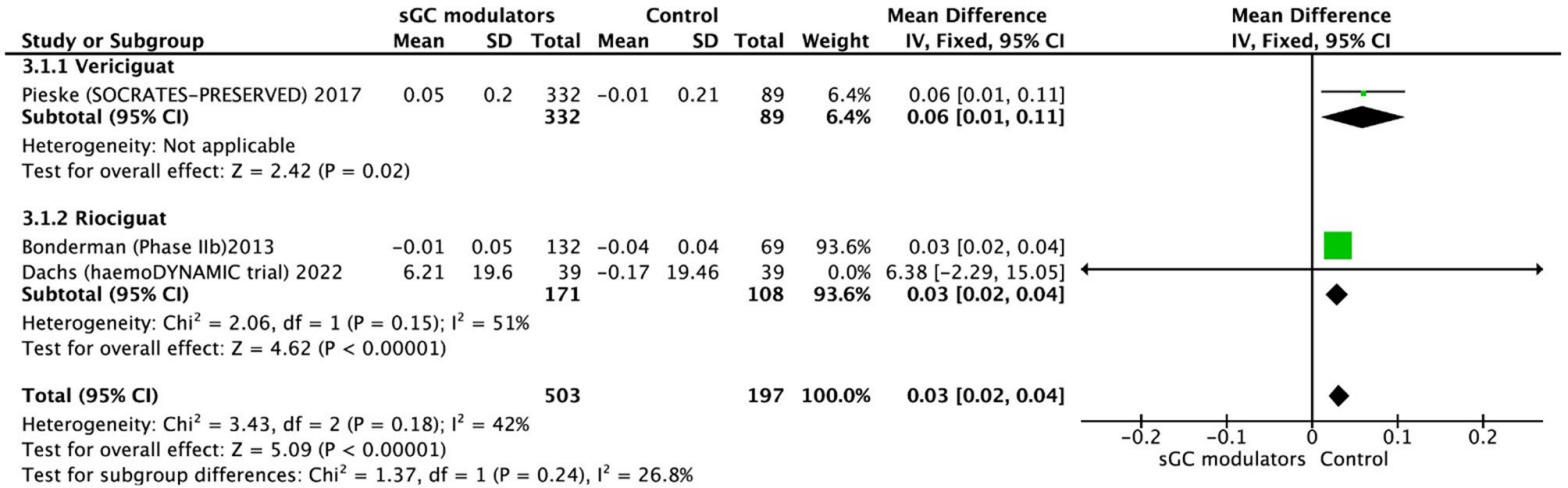
Forest plot on the change of EuroQol Group 5-Dmensional Self-report Questionnaire US index in participants with heart failure: randomized controlled trials (RCTs) that compared soluble guanylate cyclase modulators to placebo. CI = confidence interval.

In three trials, health-related quality of life was measured using “EuroQol Group 5-Dimensional Self-report Questionnaire (EQ-5D) US Index” scores. As a subgroup analysis, one study examined the health status of patients who participated in the SOCRATES-PRESERVED RCT^21^. According to the findings of studies that used the fixed-effect model, the sGC modulators significantly improved EQ-5D based quality of life in patients with HF (MD = 0.03, 95% CI 0.02–0.04, *p* < 0.001), without significant heterogeneity in three trials (I^2^ = 42%, *p* = 0.18) (Fig. 4).

**Figure 5:**
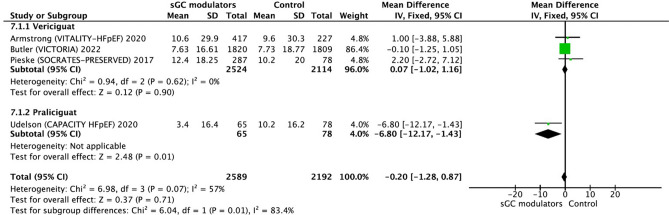
Forest plot on the change of KCCQ in participants with heart failure: randomized controlled trials (RCTs) that compared soluble guanylate cyclase modulators to placebo. CI = confidence interval.

Of note, based on the findings from studies -utilizing the fixed-effects model-, sGC modulators showed no significant alteration in the KCCQ score (− 0.20, 95% CI − 1.28 to 0.87, p = 0.71) (Fig. 5), and this was observed without significant heterogeneity (I^2^ = 57.0%, *p* = 0.07).

**Figure 6:**
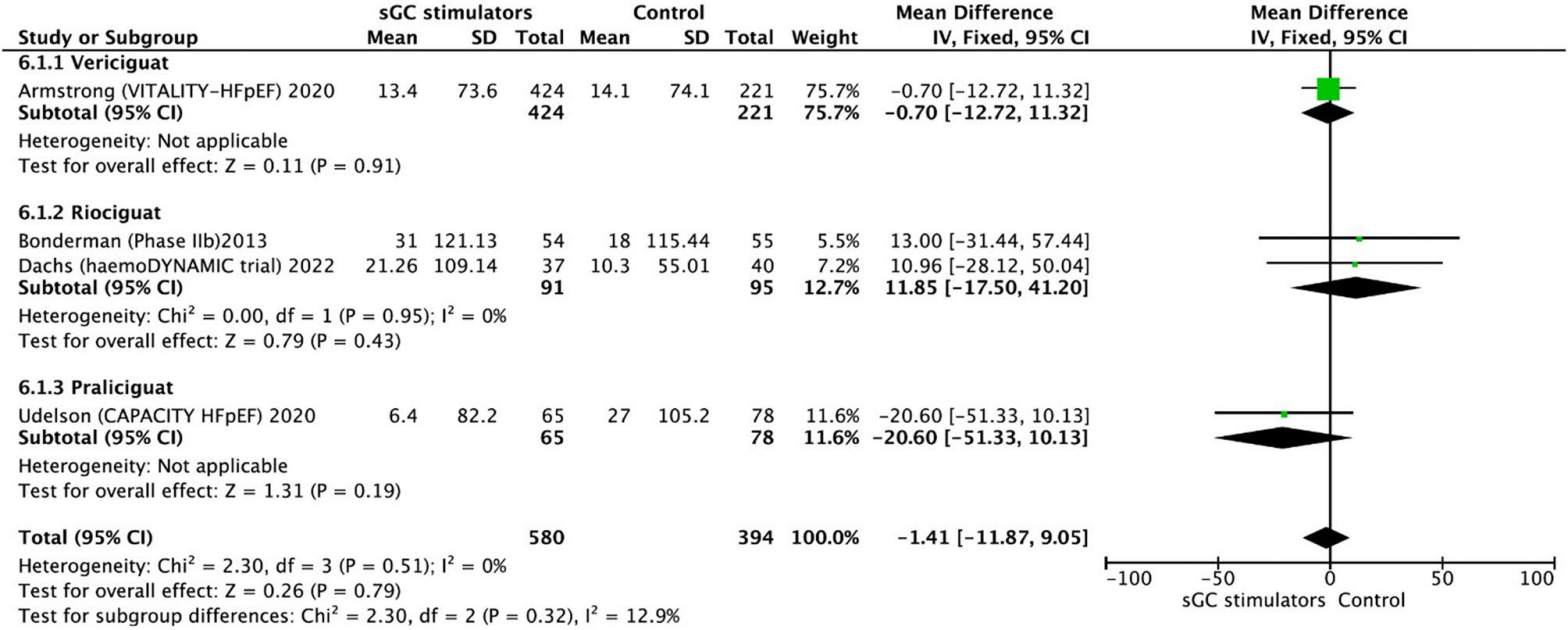
Forest plot on the change of 6-MWD in participants with heart failure: randomized controlled trials (RCTs) that compared soluble guanylate cyclase stimulators to placebo. CI = confidence interval.

The effect of sGC modulators on 6MWD was also evaluated. The meta-analysis results revealed that sGC modulators had no remarkable effect on 6-MWD in patients with HF (− 1.41, 95% CI − 11.87 to 9.05, *p* = 0.79). There was no significant heterogeneity among the studies (I^2^ = 0%, *p* = 0.51) (Fig. 6). Therefore, the analysis was carried out using the fixed-effects model.

**Figure 7:**
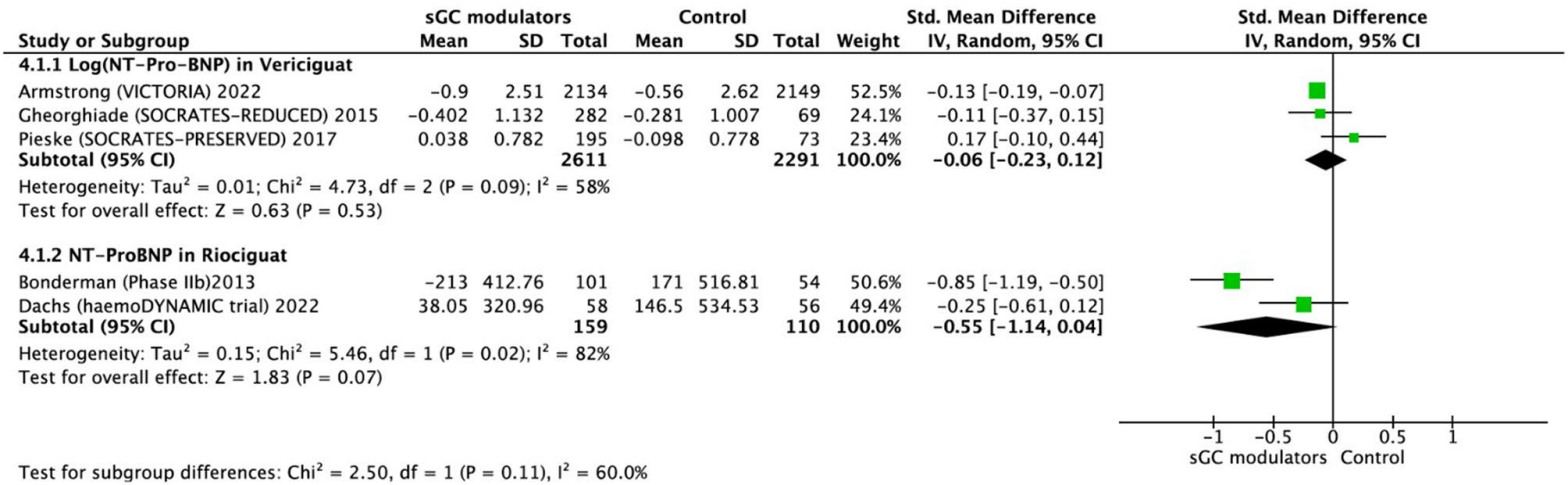
Forest plot on the change of log (NT-proBNP) and NT-proBNP in participants with heart failure: randomized controlled trials (RCTs) that compared soluble guanylate cyclase modulators to placebo. CI = confidence interval.

The effect of sGC modulators on the change of NT-proBNP from baseline was examined in five trials. The log (NTproBNP) was included in the vericiguat group, indicating that vericiguat overall did not significantly decrease log (NTproBNP) values as compared to control group (− 0.06, 95% CI − 0.23 to 0.12, *p* = 0.53), though, single large study of vericiguat in HFrEF yielded trivial change in NT-proBNP levels in relation to outcome (Fig. 7). The riociguat treatment, also ended up with similar overall trend (− 0.55; 95% CI − 1.14 to 0.04; *p* = 0.07). There was no significant heterogeneity in vericiguat group (I^2^ = 58%, *p* = 0.09). Significant heterogeneity was observed in the riociguat group (I^2^ = 82%, *p* = 0.02). (Fig. 7).

**Figure 8:**
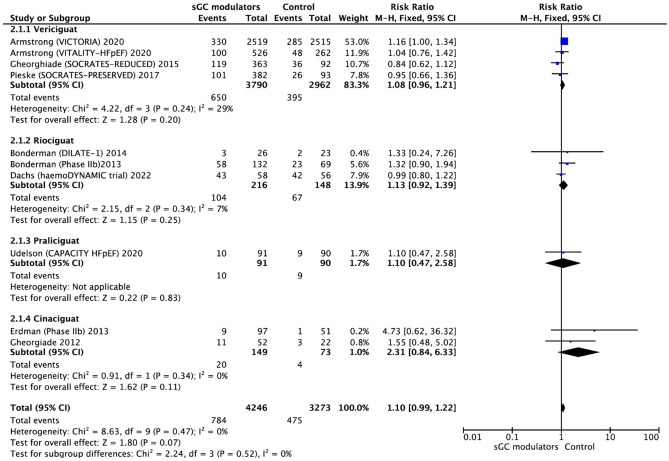
Forest plot on the occurrence of SAEs in participants with heart failure: randomized controlled trials (RCTs) that compared soluble guanylate cyclase modulators to placebo. CI = confidence interval.

The rate of drug related serious adverse events of therapeutic significance was calculated using ten RCTs involving 7,526 participants (4253 sGC modulators and 3273 placebo), that included ventricular tachycardia, cardiac failure, syncope, peripheral edema, pulmonary edema, hypotension, decreased cardiac output, headache, and pulmonary hemorrhage. The use of sGC modulators in HF was associated with a trend towards a significant difference in the incidence of SAEs compared to placebo (RR = 1.10, 95% CI 0.99–1.22, *p* = 0.07) (Fig. 8).

**Figure 9:**
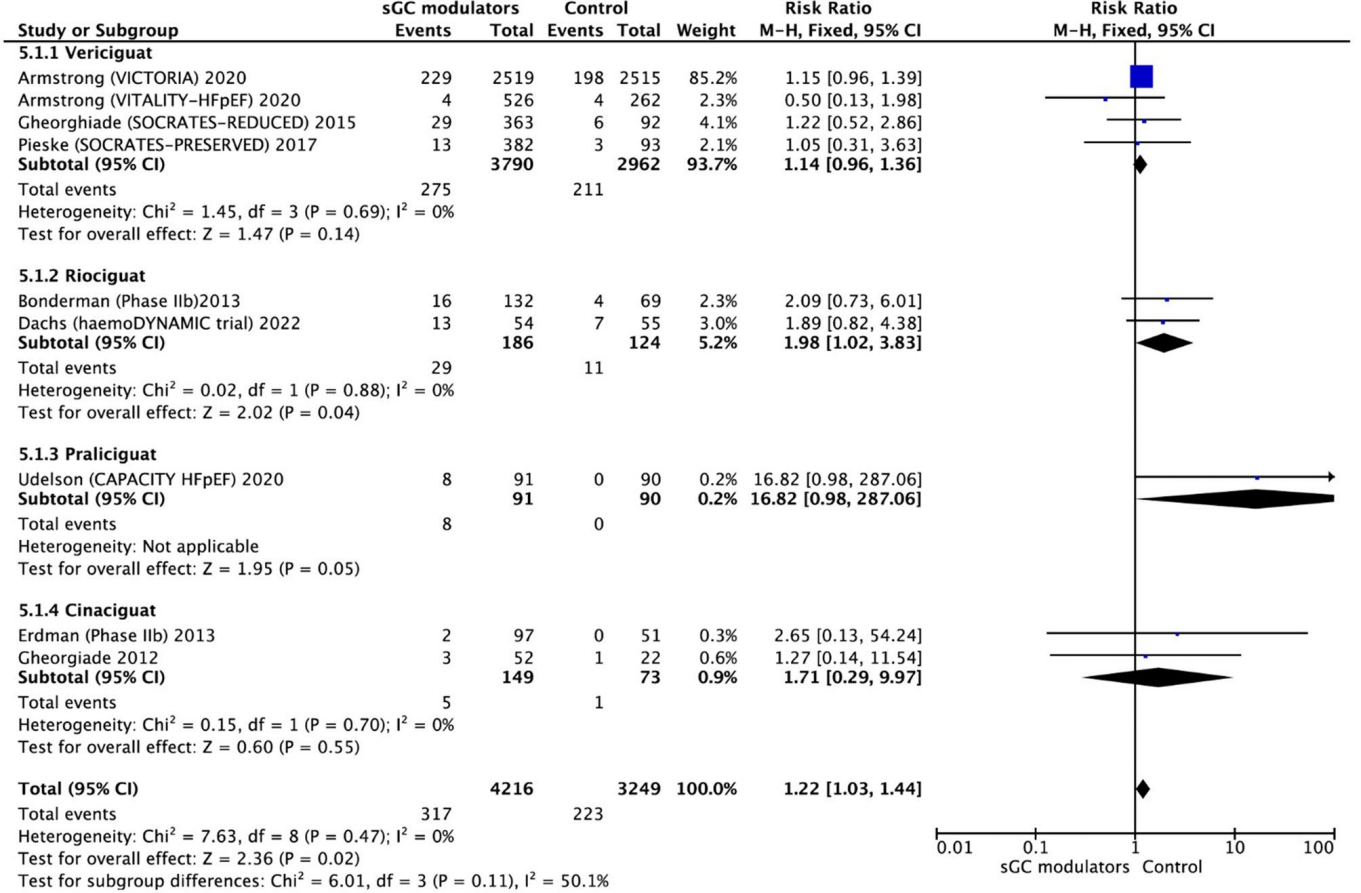
Forest plot on the occurrence of hypotension in participants with heart failure: Effect for RCTs comparing soluble guanylate cyclase modulators to placebo. CI = confidence interval.

It should also be underlined that there has been a significant increase in the incidence of hypotension (RR = 1.22, 95% CI 1.03–1.43, *p* = 0.02) (Fig. 9). There was no significant heterogeneity SAEs and hypotension, respectively (I^2^ = 0.0%, *p* = 0.47; I^2^ = 0.0%, *p* = 0.47). Based on the fixed effect model, four RCTs of vericiguat found a RR of SAEs 1.08 (95% CI 0.96–1.21, *p* = 0.20) with no signs of heterogeneity (I^2^ = 29%, *p* = 0.24). Other three studies analyzed the results of riociguat and found a RR of 1.13 (95% CI 0.92–1.39, *p* = 0.25) without significant heterogeneity (I^2^ = 7%, *p* = 0.34). The RR of participants who used praliciguat and cinaciguat were 1.10 (95% CI 0.47–2.58, *p* = 0.83) and 2.31 (95% CI 0.84–6.33, *p* = 0.11), respectively. Praliciguat was tested only in one study, therefore heterogeneity was not calculated. There was no significant heterogeneity for cinaciguat (I^2^ = 0.0%, *p* = 0.34).

**Figure 10:**
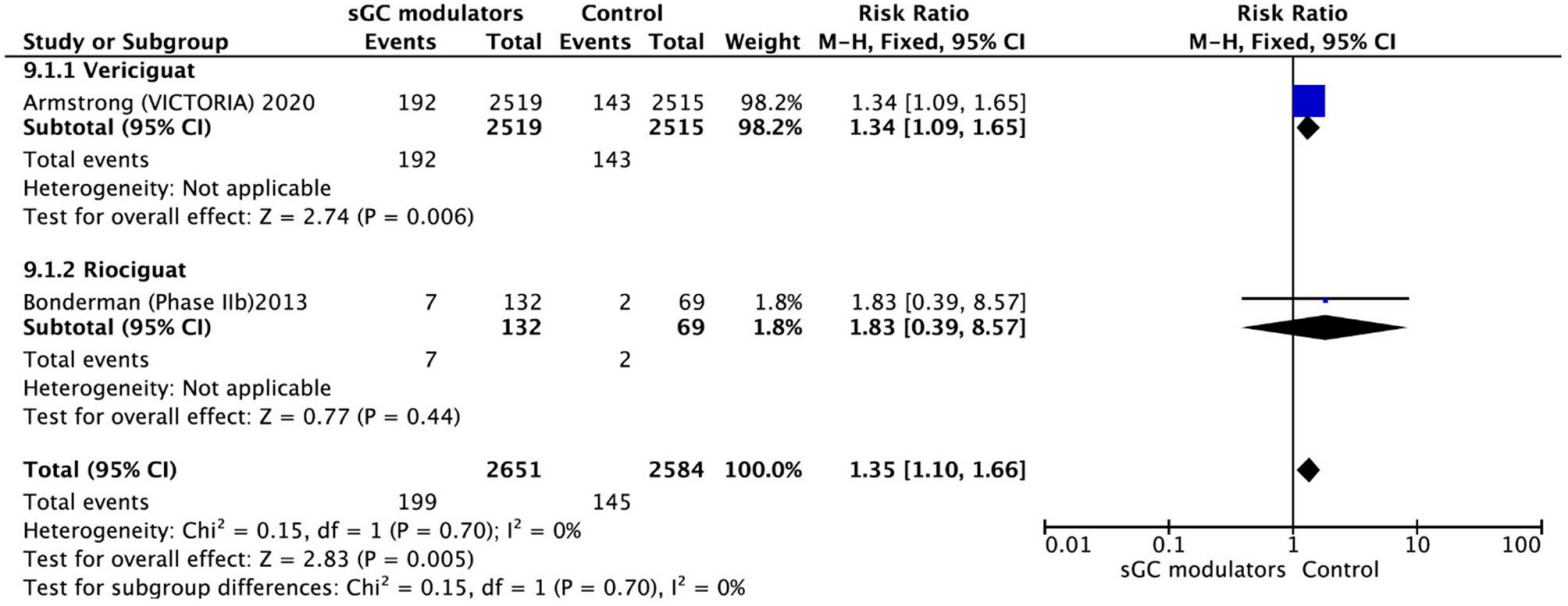
Forest plot on the occurrence of anemia in participants with heart failure: Effect for RCTs comparing soluble guanylate cyclase modulators to placebo. CI = confidence interval.

The use of sGC modulators (vericiguat and riociguat) in HF and the risk of anemia were evaluated with data from two eligible studies^18,22^. The use of sGC modulators in HF was associated with a significantly increased risk of anemia compared to placebo (RR = 1.35, 95% CI 1.10–1.66, *p* = 0.005) (Fig. 10). There was no significant heterogeneity between studies and the analysis was performed using the fixed-effect model (I^2^ = 0.0%, *p* = 0.70).

### Publication bias

To assess the possibility of publishing bias in mortality, SAEs, hypotension and 6MWD, funnel plots, the Egger test, and Begg’s test were used (Figs. S1–S4). We found no evidence of possible publication bias in the evaluation. Meanwhile, due to inadequate number of trials, the publication bias for the improvement in EQ-5D US index score, NT-proBNP, anemia, and KCCQ was not checked.

## Discussion

As an intracellular actor, cyclic guanosine-3ʹ,5ʹ monophosphate, generated by sGC out of its triphosphate version, is a second messenger which plays a significant role in physiology^28^. However, NO–sGC pathway is known to decay in HF and several agents as sGC modulators have been developed and tested in different clinical settings of HF^3^. Of note, sGC modulators differ from each other by mode of action at the receptor level. Vericiguat, riociguat, and praliciguat are known as sGC stimulators and cinaciguat as a sGC activator, and all molecules can be grouped as sGC modulators^4^. This meta-analysis designated that sGC modulators did not yield any mortality benefit in HF patients compared to placebo, and this finding was comparable to Moghaddam et al., though the authors did not mention change in NTpro-BNP levels and EQ-5D in comparison to KCCQ^29^. Of note, it was shown that vericiguat yielded benefit regarding composite outcome of cardiovascular death and HF related hospitalization in HFrEF patients with recent hospitalization. However, the sole driver of benefit was related to decreased HF related hospitalization, which has recently been linked to trivial change in NT-proBNP levels via vericiguat compared to placebo^26^. Although, there was overall neutral effect of both vericiguat and riociguat onto NT-proBNP levels, there were eye-catching differences, though, not proven statistically, as such HFrEF and HFpEF phenotypes, respond differently to sGC modulation. Patients with HFrEF tended to respond by slightly decreasing NTproBNP levels, whereas, HFpEF phenotype has a tendency to respond either by neutral or increasing levels. This was contrary to a previous analysis of Zheng et al. in which the authors reported that there was a decrease in NTproBNP levels with riociguat, but not with vericiguat^30^. Of note, VICTORIA trial had not been published at the time of that analysis^22^. In this analysis, reported sequential analysis of NTproBNP in the VICTORIA trial was included by log transformation, and there was a modest difference between vericiguat versus placebo, and this finding was reported to be modestly related to primary outcome, mainly driven by decreased HF hospitalization^26^. However, combining other trials of vericiguat ended up with a numerically lower but nonsignificant decrease in NTproBNP. Herein, the difference might be confounded by HFpEF phenotype, in which median NTproBNP levels were much lower and prone to several confounders including obesity.

Regarding quality-of-life measures, there was a neutral effect of sGC modulation with regard to KCCQ, though, there was a significant improvement with EQ-5D in this analysis. This neutral finding with KCCQ was partly mentioned for HFpEF population in Moghaddam et al.^29^, though, the large data designating no effect of vericiguat in HF with reduced ejection fraction was lacking in that analysis^27^. Hence, it is safe to conclude that sGC modulation, irrespective of HF phenotype and the agent, does not improve KCCQ scores in HF patients.

Of note, to the best of our knowledge, there is no analysis specifically considering two discrete scores, namely EQ-5D and KCCQ. Herein, we found in our analysis containing both HF phenotypes that EQ-5D scores were significantly and uniformly improved, and this finding was confirmatory to previous analysis, which only checked for change of EQ-5D^30^. However, in our analysis, KCCQ scores were also considered, and it was shown that KCCQ scores did not change by sGc modulation in HF patients, irrespective of phenotype, confirming previous analyses^5,29,31^. Hence, KCCQ seems to miss specific domains with regard to observed clinical benefit of sGc modulation in terms of HF related hospitalization, at least in the largest trial^22^. The difference between KCCQ and EQ-5D might be based on the notion that the KCCQ is disease-specific and focuses on HF symptoms and quality of life, while the EQ-5D is rather a generic questionnaire that measures health-related quality of life across different dimensions including health care utilities^31^. Hence, the benefit of sGC modulation might be related to other domains of quality of life, such as healthcare utilities, and the upcoming studies should consider this differential impact thoroughly.

Of note, a recent meta-analysis by Thakker et al. evaluating the effect of sGC solely in HFpEF phenotype reported no significant difference in 6MWD, KCCQ and SAEs^31^. There was no improvement in 6MWD with sGC modulation compared to placebo in this analysis as well confirming previous analyses^29,30^. However, regarding safety, our findings were contrary to the previous two meta-analyses which designated no difference in SAEs between sGC modulation and the placebo groups^29,30^. In the context of SAEs, particularly for hypotension, there was a considerable risk with each of sGC modulators compared to placebo. However, 2018 analysis seems to focus only on sGC stimulators, lacking the largest and the most contemporary data. On the other hand, 2022 analysis seems only to get concentrated on the effect of sGc stimulators in HFpEF patients and concluded that there was no negative signal regarding SAEs, though, efficacy was also neutral. In this current analysis, considering all the available HF phenotypes, it was noted that there was a trend towards a significant difference in SAEs, particularly a significant increase in the incidence of hypotension and anemia. To the best of our knowledge, increased risk of anemia in association with sGc modulation has not been reported elsewhere before. Of note, hemoglobin data were not extensively reported in most of the trials; hence, further analyses are required as anemia is an important contributor to prognosis.

It is necessary to mention and emphasize some of the noteworthy limitations of this study. First, there might be more differences than similarities concerning sGc modulator agents. However, sGc modulation is not a novel term, and it was introduced some years ago. Besides, the potential impact of molecular differences is not well studied. Secondly, there are phenotypic and outcome-based differences in trial designs yielding heterogeneity, though some domains were calculated not to have significant heterogeneity and bias, including data for all-cause mortality and SAEs. Hence, we think the absence of mortality benefits along with consistent findings of a trend towards a significant difference in SAEs, particularly hypotension, and, to some extent, anemia deserve to be thoroughly considered. Of note, NT-proBNP levels are overall not distinguishingly affected by sGc modulation, though there is a modest signal that it might relate to outcome. However, the HF phenotype might have confounded the plausibility of the results. It is also worth noting that the majority of the included studies did not involve patients treated with SGLT-2 inhibitors (SGLT-2i), which have been demonstrated to be highly effective across the LVEF spectrum in HF treatment. Given the proven efficacy of SGLT-2i in the contemporary management of HF, there is notable uncertainty regarding the applicability of our meta-analysis results to HF patients currently receiving SGLT-2i treatment.

## Conclusion

In conclusion, sGC modulation in HF seems not to appear to remarkably improve all-cause mortality. However, the outcomes of our investigation demonstrate that sGC modulators confer advantages regarding EQ-5D-based quality of life for individuals suffering from HF. Furthermore, it is associated with a trend towards a significant difference in SAEs, particularly an increased incidence of hypotension and anemia. Taken together, the overall effect is not driven by either change in NTproBNP or HF-related quality-of-life measures, it remains established that potential benefit of sGC modulation might be based on other parameters such as generic quality-of-life measures, which might help explain the benefit regarding HF-related hospitalization.

### Supplementary Information

Supplementary Information.

## Data Availability

The datasets used and/or analyzed in this study are available upon reasonable request from the corresponding author.
